# Supplementary material for: Does the sex difference in competitiveness decrease in selective sub-populations? A test with intercollegiate distance runners
Source: PeerJ. 2015 Apr 21;3:e884. doi: 10.7717/peerj.884 (PMC4411483; doi:10.7717/peerj.884)
Supplement: Supplemental Information 2 [file peerj-03-884-s002.pdf]

Supplemental Information:
Internal Consistency and Factor Structure of MOMS and SOQ sub-scales

This supplementary section has three purposes: 1) Test the internal consistency reliability of the SOQ and MOMS sub-scales, as defined by (Gill & Deeter, 1988; Masters, Ogles & Jolton, 1993), respectively; 2) Test the relative factor structure of a principal component extraction, and subsequent varimax rotation, against the original MOMS and SOQ sub-scales; and 3) Compare male and female sub-groups on #1 and #2 above, to ensure that the scales' internal consistency and factor structure are comparable across sexes so that mean difference comparisons made in this article are valid.

Method

The assessment of internal consistency reliability of the measured scales was accomplished by coefficient alpha calculations on the overall sample, as well as male and female sub groupings. Factor structure was assessed by examination of post-rotation (varimax) eigenvalues of extracted principal components. This allowed examination of both similar spread of variance across factors, as well as percentages of variance accounted by the solution. This procedure was run on the overall sample, as well as male and female sub groupings.

Results

Comparisons of coefficient alpha across sub-scales and sub-groups can be found in Table S1.

Table S1.
Coefficient alpha of MOMS and SOQ measures, by sub-group.

	Sub-scale (# of items)	Overall	Male	Female
MOMS				
	Affiliation (6)	.881	.878	.876
	Competition (4)	.830	.806	.822
	Psychological coping (9)	.918	.923	.908
	Self-esteem (8)	.857	.867	.846
	Health (6)	.884	.892	.865
	Life meaning (7)	.895	.887	.901
	Goal achievement (6)	.840	.821	.856
	Recognition (6)	.887	.884	.892
	Weight (4)	.847	.800	.861
SOQ				
	Win orientation (6)	.851	.839	.850
	Competitiveness (13)	.900	.899	.888
	Goal orientation (6)	.813	.829	.791

Table S1 indicates that sub-scale internal consistency was very high across both the MOMS and SOQ sub-scales, and it remained high when examined for male and female sub-groups. Although not as detailed as the next analysis, this pattern can also be viewed as preliminary evidence that the factor structure held across male and female sub-groups as well.

Eigenvalues and variance accounted for by the sub-scales can be found in Table S2.

Table S2.

Post-rotation eigenvalues and accounted variance for MOMS and SOQ measures, by sub-group.

	Factor	Overall	Male	Female
MOMS				
	1	6.60	7.17	6.71
	2	4.73	4.88	5.46
	3	4.20	4.74	4.85
	4	4.20	4.52	4.83
	5	3.98	4.25	4.36
	6	3.89	4.12	3.49
	7	3.55	2.57	3.24
	8	2.73	2.40	2.41
	9	2.41	2.17	2.30
	%var	*.648*	*.658*	*.672*
SOQ				
	1	4.02	2.99	3.87
	2	3.20	2.92	3.41
	3	2.78	2.74	2.50
	%var	*.400*	*.346*	*.391*

Note that Table S2 is formatted similarly as Table S1, only the MOMS and SOQ sub-scale labels have been replaced by factor numbers. This numbering corresponds to the order of extracted factors; it does not correspond with the rows in Table S1.

Several conclusions can be drawn from the eigenvalues in Table S2. The overall factor patterns for both the MOMS and SOQ measures remain similar across male and female sub-groups. The relative percentage of variance extracted remained similar across male and female sub-groups but differed between the MOMS (range = .648 - .672) and the SOQ (range = .346 - .400). This was almost certainly due to the MOMS being comprised of nine factors versus the SOQ's three factors.

Conclusions

Overall, these reliability and factor analyses establish both the internal consistency and the factor structure of the originally defined MOMS and SOQ measures, as well as demonstrate that internal consistency and factor structure are similar across male and female sub-groups.

References

Gill DL, Deeter TE. 1988. Development of the sport orientation questionnaire. *Research Quarterly for Exercise and Sport* 59:191–202.

Masters KS, Ogles BM, Jolton JA. 1993. The development of an instrument to measure motivation for marathon running: The Motivations of Marathoners Scales (MOMS). *Research Quarterly for Exercise and Sport* 64:134–143.
